# Progressive Paralysis of the Insane

**Published:** 1868-05-01

**Authors:** A. W. Bosworth

**Affiliations:** Paris, France. Thesis for the Degree of M.D., Presented to the Faculty of the Rush Medical College, Chicago


CHICAGO MEDICAL JOURNAL.

*Vol. XXV.—MAY* 1, 1868.—*No.* 9.

### PROGRESSIVE PARALYSIS OF THE INSANE.

(PARALYSIE GÉNÉRALE DES ALIÉNÉS, OF THE FRENCH.)

BY A. W. BOSWORTH, M.A., PARIS, FRANCE.

Thesis for the Degree of M.D., Presented to the Faculty of the Rush Medical College, Chicago.

Of cerebral diseases, this is one of the most important, most common, and, in regard to its symptoms, march, termination and pathological anatomy, one of the best characterized.

Although asylums contain a large number of this class of patients, and notwithstanding it has been described as a distinct disease for the last forty-five years, yet to-day we see many practitioners unacquainted with it, unless they have followed a special service in some large town.

Not being able, in an ordinary thesis, to dwell on its history, we can only briefly mention some of the different ways in which it has been mentioned by the principal authors.

Esquirol, 1805, remarked the incurability of insanity, when complicated with palsy. In 1844, in his article on Dementia, he considered the palsy as a complication of insanity, and not as a distinct affection.

Bayle, 1822, was the first to admit that the intellectual derangement and the palsy marched simultaneously; and considering the disease due to chronic inflammation of the arachnoid, he styled it "Chronic Arachnitis," and divided it into three periods: 1st, Congestion, ambitious monomania, with incomplete palsy. 2d, Mania, inflammation of the meninges. 3d, Dementia, the general palsy being more salient, due to serous exudation.

Georget regarded palsy as consecutive to insanity.

Calmeil, 1820, gave a complete description of the disease, and asserted that the principal anatomical lesion was not an inflammation of the meninges, with its reaction on the cortical substance of the brain, but an inflammation of the cortical part itself; that the palsy was not owing to compression produced by the serous effusion on the brain, but to the disorganization of the gray substance.

Parchappe, 1838, gives the softening of the cortical part as pathognomonic of the disease, dementia paralytica—folie paralytique—having for its essential character the simultaneous lesion of movement and of intelligence.

Baillarger, 1846, endeavored to prove that the lesion of movement, and especially of speech, precedes the symptoms of delirium. He considers the disease neither as a complication of dementia, nor a particular form of mental alienation; but as a special and independent malady, especially characterized by lesion of the movements, which may be complicated with secondary mental alienation, or may exist alone.

M. Marcet, 1862, regards the disease as being constituted by four distinct associated elements, viz.: 1st, Disorder of motility. 2d, Intellectual weakness. 3d, Delirium, characterized, in the majority of cases, by exaltation and ambitious ideas. 4th, As a constant organic lesion, the adhesion of the meninges to the cortical layer of the circumvolutions.

Mr. Fabret, junior, gives the following conclusions: 1st, Dementia paralytica is distinct from all other general palsies. 2nd, General palsy does not occur among the insane who have been a long time in asylums; it is of another nature, and depends on another disease; ex., softening of the brain, apoplexy, cerebral tumor. 3d, The insane, who die paralytic in asylums, already presented symptoms of palsy on entering, or soon after; and, in every case, died within three or four years after admission.

Some authors admit two distinct kinds: 1st, General palsy, with insanity. 2d, General palsy, without insanity.

It is this last form, which all authors do not admit. The two forms have been confounded by some, under the name of General Progressive Palsy. As a distinction between the two, the first is considered incurable; the second, less dangerous, and often cured. This distinction is contested, as all authors do not admit the latter form. Most authors, however, agree that the disease is incurable, and believe that the examples given as recoveries are only remissions.

This malady being frequent, serious, incurable, attacking especially man, and, when in the bloom of life, conducting him through sad stages of degradation to an untimely grave, has caused me to wish to study it, and to here use it as my theme; not expecting, however, to be able to advance the knowledge of the profession thereby.

It is anatomically distinguished by chronic congestion of the cortical substance, and adhesion of the meninges. Its symptoms are: disorder of motility, dementia, and delirium, often of an ambitious tendency.

It is of great importance to be able to establish the first indications of this disease, not only in a medico-legal point of view, but for the reputation of the physician, who, if he does not recognize the insidious beginning, will have the mortification of seeing his reputation compromised; and also for the patient, who, if left unguided, may soon be dangerous—hazarding life, fortune, and the honor of himself and his fellow beings.

As precursory symptoms, many months before its invasion, arrive light cerebral congestions, which cause redness of the face, trouble of vision, heaviness of the head. An intense redness, some times with pain, has been noticed to occupy one or both ears. These symptoms are characterized by their short duration, and may return after each meal, or between them, at irregular hours of the day and night. Before, during or after the congestion, there may be more or less cephalalgia, with epistaxis. At the same time, appear a particular disposition of the mind, changement of character and manners, which can not be accounted for by friends. There is a perversion of the moral faculties, no longer harmoniously performing their functions; persons or things heretofore cherished by the patient, become, for the moment, completely of no account; at one time, he is seen contradicting all, then again acquiescing in every respect; in a short time, sustaining, with the same obstinacy, contradictory opinions. In three-fourths of the cases, irritability is intensified; movements of impatience, anger, and violence, may be observed from the slightest cause. On the contrary, some times remarkable placidity is evident; disgust for serious occupations; apathy for amusement; or, the patient, while yet attending to his daily vocations with customary assiduity, although of honorable, pure, religious habits heretofore, may now commit indelicate, scandalous acts of dishonesty and debauchery.

As the period of incubation is so insidious, all that concerns the moral condition of the patient, should especially demand the physician's attention. As a great many persons predisposed to mental disease, frequently manifest strange peculiarities, if they are carefully examined, we find characteristics of noted opposition, inconsistent with age and education. Some are gay, eccentric, running to and fro for every mundane joy; others are reserved, melancholic, secluded, shedding involuntary tears, succeeded some times by bursts of gaiety—soon, however, to be sadly deplored. With some, vanity or humility, timidity or boldness, want of order, in even the most trifling affairs; scrupulous, some times, to an excessive degree, or presenting stolid indifference. Often vacillating, their affections, desires, dislikes, degenerate into ardent passions, causing them to pass rapidly from enthusiasm to discouragement, or *vice versa*.

While friends are now being perplexed, not perceiving the true nature of conduct to them inexplicable, the precursory period, short or long — after M. Brierre de Boisimont being able to continue six or seven years — intensifying passes into the

### First Stage.

Although the precursors of this period are sufficiently uniform, yet when the disease is at its present stage it is so varible that with reason do we think, Messrs. Jules Favre, Limas, Marcé, etc., are well authorized in admitting four distinct varieties; in two of which, the paralytical and congestive forms, predominate physical symptoms; in the other two, the expansive and melancholic, are clearly evident psychical symptoms.

*Expansive Form.*—This is considered the most common. After a precursory period of various duration, the patient, now irritable and violent, becomes more active than is usual. Proud, enterprising, not finding enough in his profession to satisfy his devouring activity, he exaggerates it, or undertakes several vocations at the same time, pushing them to a colossal degree; but, as has been truly remarked, this activity is soon only sustained by words. All his numerous projects rest at a mere beginning. His ambitious ideas are not only absurd, but contradictory. His acts are often singular, droll, dangerous, or even criminal. We now see that his memory and reasoning faculties are weakened. He forgets what he has done or intended to do a few hours before; strongly declares that he has not eaten for a day or two, when but a few hours preceding he ate a hearty meal. With this forgetfulness of the present, it is not uncommon to see coincide a perfect remembrance of facts that occurred years ago. His mind may yet retain brilliant parts, but generally it takes the wrong direction. Surrounding persons see that his intelligence has diminished; the mind has no longer its ordinary firmness and precision, but there are deficiencies, reveries, forgetfulness, causing the patient to get confused in a conversation a little prolonged; he can no longer explain a subject, or an important fact, with his heretofore customary clearness, losing himself either in the details or in the conclusions.

Symptoms of organic excitation are not less accentuated. With inability to remain motionless, they are obliged to yield, and are seen walking, running, leaving their abodes, and roaming through the streets or fields, undressing themselves and lying down in the open air. Now impelled to mental and physical activity, they are rendered bold, never doubting to fail in an undertaking. Even the most avaricious rush headlong into the most hazardous speculations; embellish their houses without having money to pay; make numerous presents; in every way profusely spend, and far surpass their incomes or resources. With this irregularity are seen various excesses, such as being given to alcoholic drink, which certainly aggravates their sad condition; an erotic passion, becoming so unsatiable that they pass all bounds to gratify it; thieving in a peculiar manner, apparently without design. The malady advancing now brings to the observer all the symptoms of true maniacal delirium of an ambitious nature, which pertain to making of himself an *Ego*. His contradictions are not revolting to him; he does not strive to reconcile or prove the ideas advanced; he relates, at the same moment, his real and his imaginary life, without endeavoring to make them co-ordinate. He sees no bounds to his ambition; he is at once pope, emperor, king, or president of a state; he has numerous servants, houses, castles, treasure without end; they cause to descend on the persons about them, on the physician himself, dignities of all grades; it costs them nothing to make promises and to offer services. Sometimes they consider themselves supernatural, God himself, and heal the sick; make the blind to see, the lame to walk; create men of prodigious size. They can cause to die and resuscitate whom they choose; they make balloons the size of a large town; they have measured the sea, the surface of the earth; give feasts to thousands of persons at the same time.

Sometimes it is a mania for marriage; they are going to espouse princesses, queens; forget their own wives and children; the sun, moon, and the stars converse with them; *they* are not fools, and ask consultations from the doctor; the physician who treats them is the one insane, and they mock him to his face, and pity him, or consider him their enemy.

Their attacks of Erotomania are exaggerated to the same degree, and often lead them astray. There is another class of patients who, though not presenting this train of ambitious ideas, and not having, properly speaking, delirious conceptions, yet live in a state of happiness, supremely contented with themselves. They have pretensions to literature, poetry, strength. They think themselves vigorous, never in better health. They admire themselves, and endeavor to have others admire the beauty of their person. They spend hours in arranging their clothes properly. With a frequent smile on their face, they approach the first comer with marked cordiality, and desire to show all they possess. As the disease advances, their happiness presents a strange contrast with the physical and intellectual degradation in which they are, and without moral suffering they pass happily to the embrace of death. The ambitious monomania of paralytics is easily distinguished from ordinary ambitious monomania. This last is rare; its victim is morose and taciturn at the beginning; perceives that he is ill, fears for his reason; endeavors to repel, at first, the delirious ideas, which he considers absurd. Later, mastered by the delirium, he is in harmony with his thoughts; he assumes the position, manner, gesture, voice, of the personage he has chosen. His discourse, usually the same, is consistent with the position he believes himself designated to fulfill. General, king, or prophet, he only uses terms coinciding with his illusory dignity; majestic and dignified, he remains taciturn, not taking the first person seen for a confidant, but retains all to himself. His delirium is fixed, unchanging, and he gives proofs of a greater or less value of what he asserts. Hallucinations, rare with the paralytic, on the contrary are with him frequent.

Far different from the preceding is the petulance and stupid haughtiness of the paralytic, which changes at every instant, characterizing itself by the particular nature of the ambitious ideas, which are multiplied, absurd, and contradictory. We easily see with them that memory and reason are profoundly affected. To-day king or emperor, to-morrow pope or Divine Creator, showing no tenacity for their opinions, yielding them if contradicted; ideas as heterogeneous as the products of a volcanic eruption; laughing or crying at the same moment. Their weeping singularly presents itself; while so doing the face is pale. They regard those who surround them, not endeavoring to conceal their tears, every indication of intelligence disappearing from their countenance; we perceive that they are not conscious of their actions.

Only the idiot and the cretin thus weep.

At the same time as this excitation, intellectual weakness, and ambitious ideas are manifested, we observe trouble of motility and of sensibility, *i. e.*, difficulty of articulating sounds, cutaneous anæsthesia and inequality of the pupils.

*Imperfect Articulation.*—This arises from an irregular, convulsive contraction of the lingual, labial, and facial muscles, producing at the beginning a slight, transitory trembling of the tongue, occurring perhaps at long intervals for certain words or letters, as the letter R. Even before the patient has pronounced a word, as he opens his mouth to speak, may be observed a characteristic trembling of the orbicularis oris, and of the levatores labii superioris proprii, with even an oscillation of the lower jaw; and then with hesitation and difficulty the first syllable is pronounced. This impediment in speech presents different degrees; at the first it is difficult to be recognized; the speaking is less distinct. The patient speaks with a difficulty, evident to the physician by the trembling of the lips, by hesitation, by a false endeavor seized only by an experienced ear; or his words may be blunt, precipitated, appearing strongly guttural. At a more advanced period exists stammering, which is pathognomonic of the disease. Now by causing the poor creature to extend the tongue, we perceive its peculiar trembling, he not being able to retain its immobility for a moment. After meals, during periods of anger, with some, difficulty of speech is more evident; with others, during periods of excitation it is less marked, and only is remarkably apparent during their moments of depression. This characteristic of the disease being so variable, the physician must choose his moments of examination, and possibly will be obliged to make his patient speak several minutes, or read some difficult passage, before he will manifest this keystone to his disease.

At a more advanced period still, with some are observed continual movements of deglutition, noise of the dental arcades coming in contact during mastication, continual or interrupted grinding of the teeth at different hours of the day.

The Inequality of the Pupils, first recognized by M. Baillarger, may appear at the beginning of the disease, and at the period when the victims have arrived in the hospital, usually is easily perceived. Like other symptoms, this, at moments, is observable, and then disappears only to return at intervals more or less long. For its explanation we can here merely say that it may be found explained by M. Baillarger in the "Gazette of the Hospitals," of Paris, May 14th, 1850.

Evident it is that the idea of diagnosticating the kind of paralytical delirium by the dilatation of the pupils is not yet established. After M. Mareau (Paris, 1853), who has seen the pupillar inequality in more than half of the cases, there is an abnormal convexity of the eye; the sclerotic is bluish; the eyebrows are curiously arranged, being in more than fifty cases in a hundred, elevated on the forehead, or falling over the eyes after the manner of the hair of the upper lip.

Lesions of Motility are not less interesting to be studied in other parts of the body, especially of the inferior and superior members. M. Tardieu especially notices the alterations of motility in the superior members. Other authorities have asserted that the palsy attacked firstly the arms, then the legs; yet observation does not prove their assertions, but tends to prove that both extremities are simultaneously seized.

Formications and prickling sensations are felt in the members during the first accidents. Often it is only a simple, nocturnal weakness; fatigue, which sleep and repose do not calm.

The patients are abnormally active, being able at moments to exert great muscular energy, but their gait is heavy; they make false steps; they appear to be propelled onwards by the weight of their own body, and not apparently stopping more easily than does the momentum of an inert body. The muscular weakness, the trembling and non-coördination of movement of the thoracic members may be observed at the beginning of the disease, by causing the patient to extend his arm and hand — then is trembling evident; by giving him a glass of water to hold; by causing him to thread a needle, or to write a few words. The characteristic writing has been mentioned by various authors.

*Melancholical Variety.*—Many authors have noticed this disease to take a sorrowful form, thus remaining for a shorter or a longer period of time. The patients believe themselves dishonored, ruined; think that they have committed crimes, and are being pursued by justice, that they are destined to be imprisoned, hung, or decapitated. With them melancholia may attain a stupid state; they remain incapable of acting; dumb, not moving, refusing food; falling when raised, and believing it impossible for them to walk. During their sadness, when most lamenting, sometimes may be noticed ideas of grandeur, ambition, which truly are of importance as clearly evincing the disease.

With this melancholical delirium, a special hypochondriacal state of the mind may sometimes be seen. After M. Baillarger, in three fourths of the cases there is a special hypochondriacal delirium. This latter is recognized by the patient believing that his organs are changed or destroyed. They think they no longer have a mouth, or stomach; no more blood; their intestines are full; their food passes under their skin, into their clothes; their body is in a state of putrefaction; can no longer urinate, or go to the *garde-robe*; can not open their eyes, because they are blind. Some believe themselves dead, rest immovable, refusing food, and thus are obliged to be nourished with a tube. We should, however, remember that the above symptoms are only of great value in point of diagnosis when they are associated with trouble of motility, with inequality of the pupils, difficulty in the pronunciation, which may present themselves with various degrees of accentuation. We must not forget to obtain, if possible, the antecedents of the patient, by which will almost invariably be found a weakness of the memory that has preceded the above symptoms for several months.

M. Baillarger has remarked that general palsy rapidly marches with these patients, and that there is great tendency to gangrene of the tissues, and an adynamic state promptly mortal; yet observation proves that often the melancholic or hypochondriacal state, as well as stupidity and an inclination to suicide, may be prolonged for months.

M. Moreau, (of Tours) in 1860 declared, at the Imperial Academy of Medicine of Paris, that the hypochondriacal delirium could not be considered as a precursor of general palsy; that when it appears, the palsy has usually been affirmed or announced by other symptoms. On this point there is a difference of opinion. M. Moreau also declared that there exists between the special delirium and general palsy affinities, very intimate, if not necessary; that the special delirium borrows its semeiological value from a morbid state more general, and influencing more profoundly the organism; that this state is a general depression, the slow and progressive annihilation of the vital powers, observable among all paralytics; that the special, hypochondriacal delire, and certain other analogous forms of delirium differ in this respect; the first taking its origin from abnormal sensations, real but disfigured, transformed by a troubled intelligence; the second being the result of a morbid working of the mind, and of delirious preoccupations.

*Paralytical Form.*—The absence of delirious conceptions, and the predominance of the troubles of motility, characterize the disease. When assuming this form, M. Parchappe (page 144), who published forty-three observations, gives the following conclusions: "We may conclude that the ideas of grandeur, riches, power, exaggeration, far from being characteristic of delirium which accompanies palsy, are only found in one case in four." In another place the same author contradicts himself by saying, "General palsy is a complication of madness; a new element which associates itself with the intellectual disorders, or else a peculiar form of mental alienation. Paralytics are, above all, insane, the principal phenomenon being insanity."

Messrs. Baillarger and Prus say, "General palsy exists in a great many cases without insanity, and that then the patients die in their families, not having gone to the asylum, and thus escape observation." Trousseau, Ducheme (de Boulogne), Requin, Delaye, Grisolle, etc., admit that the disease may occur without alienation.

Messrs. Brierre (of Boismont), Marcé and Fabret contest this opinion.

Esquirol, 1816, and Georget, 1821, considered general palsy as a complication of insanity.

We will briefly mention some of the symptoms noticed in this variety. The patients themselves are conscious that their movements are gradually losing their regularity and force; that while walking they easily tremble, and are quickly fatigued. The habitual precision of the arm and hand being impaired, they find themselves unable to play the piano, sew or write. At the same time the lingual and facial muscles present their characteristic trembling, pronunciation thus becoming difficult; the pupils become unequal, and all the motility of the system is more or less affected.

If the physician is often permitted for some time to see the patient, or the persons in daily contact give attention, they will perceive transformations of the mind; that the patient is easily irritated; at one moment is very violent, and soon after may be weeping, as it were, without cause. They are fearful, easily guided by those who surround them. Their memory, for things of recent occurrence, is weakened; their intelligence is lessened; and though not always easily perceptible, yet sometimes so manifest that the patients are conscious of it, even should they continue for a time their various vocations. However little accentuated may at first be the mental lesions, later there can remain no possible doubt of the existence of the essential elements of general palsy; namely, diminution of intelligence and trouble of motility. Often, by careful attention, may be observed in the march of this form, even transient, ambitious ideas. This form may continue for months, or some years, slowly increasing in intensity; but, sometimes, in the space of a few hours, it may make such rapid progress as to necessitate the removal of the patient to an asylum. In some cases the delirium, instead of being maniacal, may assume the form of dementia.

*Congestive Form.*—Although cerebral congestion complicates all the varieties of general progressive palsy, yet, as in many cases congestions occur before other precursory accidents are sufficiently manifest to characterize the disease, this stage, for a certain class of patients, may be considered as the principal prodromic symptom. Sometimes it begins by a violent congestive attack of a convulsive or apoplectic form.

At first it is considered as an ordinary cerebral congestion; but when the first accidents of the attack have disappeared, then are seen to appear maniacal excitement and ambitious ideas. Often, however, after the congestion, reason appears to return, but the memory is left weakened, intelligence diminished, and a slight difficulty in speaking continues. These latter symptoms tend to disappear when a renewed congestion recalls them with more or less intensity. This may be repeated a greater or less number of times, and with the capillaries of the face, neck, ears, and hair injected, the patient, with more or less of the primary symptoms, passes to the second period of the disease.

### SECOND STAGE.

Whatever may have been the fears or doubts in regard to the nature of the disease with which we have been called to combat, now the intellectual weakness and the troubles of motility are sufficiently characterized to confirm all.

The actions now have no object in view. The patients go and come; displace objects without any motive; tear their clothes; undress, getting into the first bed they find; put on one garment for another; speak much to themselves; write important letters to persons unknown to them; become inattentive as to cleanliness of their person and their clothes; do not even know their most intimate friends; declare themselves unmarried, or childless, when the contrary is true.

During their paroxysms they become quarrelsome, violent, and can exert great muscular force. The intellectual faculties being so perverted, they often perform acts of indelicacy, improbity, debauchery. During these rapidly varying derangements, we perceive traces of the same delirium which characterized the first stage, usually of an ambitious tendency; yet, not unfrequently, of a melancholic or hypochondriacal nature. The troubles of motility are also very evident. The impediment in speaking is immediately seen if an attempt is made to utter certain syllables; the trembling of the limbs, now more intense, is sometimes accompanied with such a grinding of the teeth as to be heard at a distance, and even so often occurring as to wear away a part of the enamel. The muscular contractions are now so weakened and irregular that the patient has great difficulty to walk, to aid himself. The patient, if seated, raises and lets his feet heavily fall; incomplete, continued, or alternative extension and flexion of the limbs is seen; sometimes muscular contraction is so prolonged as to produce incurvation of the head, limbs, or body.

### Third, or Final Stage.

It is considered to arrive when there is an involuntary emission of the urine and fæces. At first this may occur only occasionally, but later they can retain no fœcal matter unless it be in a solid state, as the vesical and anal sphincters are paralyzed.

Although the patient may have arrived at this physical degradation, he sometimes so recuperates as to again retain for months control of the anal and vesical sphincters. The difficulty of speaking has now so far advanced as to render the patient's mutterings unintelligible, even should he make great efforts to speak distinctly. Now he remains more or less dumb, occasionally imperfectly pronouncing a word, as million, or king, sufficient to show there yet remains the primitive, special delirium. The victims trembling when on their legs, with their bodies more or less contracted, take to their beds; finally, while unable to turn themselves in bed, yet, till the last moments of their existence are they able to execute some movements with their palsied limbs, which characterizes this palsy from all others. The respiratory muscles being weakened, the degree of calorification is diminished. The disease is apyretic (Calmeil, Parchappe, etc.). The heat of the skin is not augmented unless during the short periods of congestion, more common is it to hear the patients complain of a sensation of cold, often painful in the lower extremities.

They no longer have even instinct or sentiment, and it is necessary to care for them and feed them like a child, yet their nutrition and assimilation go on with astonishing regularity. To our great surprise, notwithstanding their great weakness, when appearing to terminate their mortal career, they may again receive some unknown force, and live much longer than was anticipated.

As death must sooner or later triumph, so it does with this disease, and in various ways. As paralytics gluttonously eat, and their deglutition is accomplished more in a convulsive manner than otherwise, often they die by the sticking of too large a piece of food in the pharynx, or by penetration of nutritive material into the larynx. In cases of asphyxia occurring from such a cause, unless instantaneous aid is rendered, they directly perish. Death may occur from retention of urine or the too often repeated catheterism; from chronic diarrhœa; from incidental disease; from exhaustion; not being able to swallow, they become weak, and more and more emaciated; from the suppuration of eschars produced by contact with urinary and fœcal matter; from cerebral congestion producing coma and death.

### Duration.

It is of various length for each period. The precursory period, characterized by perversion of the moral and pathetic faculties, may long be unperceived. Some authors, regarding the precursors as symptoms, join them to the first period, which may thus become of very long duration. The first period, as given by Bayle, is distinguished by impediment of speech, intellectual weakness, agitation, and monomaniacal delirium.

M. Parchappe writes that some patients remain a long time in the first period, while others present almost immediately the symptoms of the second period; to-day the patient being solid on his inferior members; to-morrow, under the influence of an intercurrent congestion, he can not take a step without falling.

M. Marcé says that the first period with some is very long, and that they may rapidly fail in the second period; that others live six months or more in the midst of the most serious accidents of the third period.

The malady sometimes begins by a form designated by many authors as *Acute*. Although in some of these cases given as acute, the invasion may not have been so sudden as stated by the writers, the precursors not having been recognized; or, they may have had to do with a case as a relapse; or, the information concerning the antecedents may have been erroneously given, or misunderstood; yet this form appears to be strictly admissible. Here, as elsewhere, the fundamental symptoms are ambitious delirium, and difficulty of speech; yet with these symptoms are joined an intense fever, impossibility of retaining any thing in the stomach; great excitement from severe congestions and epileptiform convulsions; and with acute delirium some cases fatally terminate in two, three, or four weeks. In other cases, when all seems hastening to quick dissolution, there may occur a sudden change for the better, and the affection passes into a chronic form, which is generally the common march of the disease. When the malady takes its more natural course, it is stated by Bayle to last ten months; by Calmeil, thirteen months; by M. Parchappe, twenty-three months. All authors agree in admitting that many cases have been prolonged three or four years by good hygienic and medical treatment, when the disease remained free from complication. Some exceptional cases are given with a duration of six, seven, or eight years. The principal cause of the great variation of the duration of this disease are its remissions, which have often been taken by the inexperienced for radical recovery. This suspension of bad symptoms, especially occurring during the first and second periods, must not lead the physician astray, nor cause him to brighten too much the desponding hopes of anxious friends. Let him remember that when he perceives the difficulty of speech diminishing, ambitious ideas vanishing, excitation yielding to tranquillity, weakness being supplanted by strength, that it is like the repose of a volcano preparing for another eruption; for sooner or later they will return to full, manifest possession of their victim. Usually the intellectual and physical symptoms diminish at the same time. Sometimes, however, only the mental faculties remain more or less impaired, the lesions of motility and the impediment of speech having nearly or quite disappeared. Now the patient appears well, with the exception of an occasional ambitious idea, and that his intelligence is a little impaired. He declares that he has been ill, but maintains that his reasoning powers were not dethroned, and that he no longer has need of friendly sympathy or medical treatment. With them, the experienced understanding of the well instructed physician will perceive that, in spite of their patients' assertions, their intelligence is still impaired, remains abnormal; that they are less energetic, are occupied with trifling affairs, easily influenced; without difficulty are they led by their passions or persuaded by others to excesses of various kinds.

